# Quorum Quenching in Culturable Phyllosphere Bacteria from Tobacco

**DOI:** 10.3390/ijms140714607

**Published:** 2013-07-12

**Authors:** Anzhou Ma, Di Lv, Xuliang Zhuang, Guoqiang Zhuang

**Affiliations:** 1 Research Center for Eco-Environmental Sciences, Chinese Academy of Sciences, Beijing 100085, China; E-Mails: azma@rcees.ac.cn (A.M.); xlzhuang@rcees.ac.cn (X.Z.); 2 Insitute of Microbiology, Chinese Academy of Sciences, Beijing 100101, China; E-Mail: lariena@126.com

**Keywords:** quorum quenching, bacteria, phyllosphere, biosassay, *N*-acyl-homoserine lactone

## Abstract

Many Gram-negative plant pathogenic bacteria employ a *N*-acylhomoserine lactone (AHL)-based quorum sensing (QS) system to regulate their virulence traits. A sustainable biocontrol strategy has been developed using quorum quenching (QQ) bacteria to interfere with QS and protect plants from pathogens. Here, the prevalence and the diversity of QQ strains inhabiting tobacco leaf surfaces were explored. A total of 1177 leaf-associated isolates were screened for their ability to disrupt AHL-mediated QS, using the biosensor *Chromobacterium violaceum* CV026. One hundred and sixty-eight strains (14%) are capable of interfering with AHL activity. Among these, 106 strains (63%) of the culturable quenchers can enzymatically degrade AHL molecules, while the remaining strains might use other QS inhibitors to interrupt the chemical communication. Moreover, almost 79% of the QQ strains capable of inactivating AHLs enzymatically have lactonase activity. Further phylogenetic analysis based on 16S rDNA revealed that the leaf-associated QQ bacteria can be classified as *Bacillus* sp., *Acinetobacter* sp., *Lysinibacillus* sp., *Serratia* sp., *Pseudomonas* sp., and *Myroides* sp. The naturally occurring diversity of bacterial quenchers might provide opportunities to use them as effective biocontrol reagents for suppressing plant pathogen *in situ*.

## 1. Introduction

The form of microbial chemical communication known as quorum sensing (QS) occurs via specific diffusible signal molecules [1]. By using a range of small, diffusible signal molecules, QS bacteria regulate a multitude of functions, including the production of virulence factors, bacterial motility, and symbiosis [2]. *N*-acyl-homoserine lactones (AHLs) are one of the most extensively studied signal molecules [3]. Currently, many Gram-negative plant-associated bacteria pathogens have been reported to regulate their virulence by AHL-based QS [4]. These plant pathogenic bacteria fall within a large number of species among the *Pseudomonas*, *Pectobacterium*, *Ralstonia*, and *Pantoea* [5–8]. Interference of the communication system used by these plant pathogens has been developed as a more recent strategy to suppress the expression of virulence, and abolish bacterial infection. The processes that interfere with QS have been generally termed as “quorum quenching” (QQ) [9]. The biocontrol strategies based on QQ mechanisms seem to be sustainable methods because they have a more limited selective pressure for microbial survival than biocide treatments [10].

QQ can be achieved by enzymatic degradation of the AHL signals. In the laboratory, transgenic plants expressing AHL-degradation enzyme showed an elevated protection from the plant pathogen *Erwinia carotovora* [11]. However, large-scale application of the genetically modified plant in the field has potential ecological risks, and negative public opinions also hinder the acceptance of transgenic crops [12]. The use of bacteria, that are naturally capable of degrading AHL to control plant pathogens, is considered as a more suitable approach [10,13]. AHL-degrading bacteria have been collected from various soil samples [11]. Phyllosphere, an important habitat for microorganisms, should deserve research attention as a resource of quenching bacteria [14]. Notably, they have the ability to reside within the same ecological niche as their pathogenic counterparts, which would protect plant effectively [15]. However, the native diversity and abundance of AHL-degrading phyllosphere bacterial populations are far less documented, particularly with regard to knowledge of their ecological roles.

Nevertheless, a systematic understanding of leaf-dwelling QQ bacteria should help to highlight the importance of the interactions within phyllosphere bacteria communities. Additionally, this knowledge will broaden biotechnological research in the field of the biocontrol of plant diseases, based on AHL-degrading phyllosphere bacterial resources. Therefore, the present study focused on surveying the presence and prevalence of cultivable bacteria, inhabiting leaves, capable of interfering with AHL-mediated QS systems. Leaf-associated strains were collected from the tobacco phyllosphere using a culture-based method, and the potential AHL-degrading capacity was identified using bioassay. An in-depth analysis, based on the 16S rRNA gene, was then performed to gain insight into the phylogenetic profiles of AHL-degrading phyllosphere bacteria.

## 2. Results and Discussion

### 2.1. Isolation of Bacteria with QQ Activity

**Figure 1:**
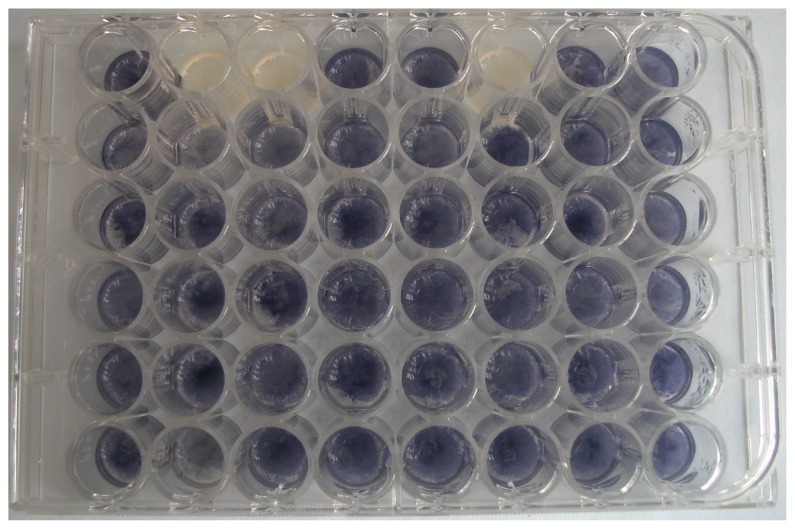
Identification of bacteria isolated from tobacco phyllosphere. Each tested isolate was incubated with 10 μM *N*-(3-oxohexanoyl)-l-homoserine lactone (3OC6-HSL) for 24 h after which the residual AHL was detected by *C. violaceum* CV026. A purple violacein indicates the presence of AHL.

**Figure 2:**
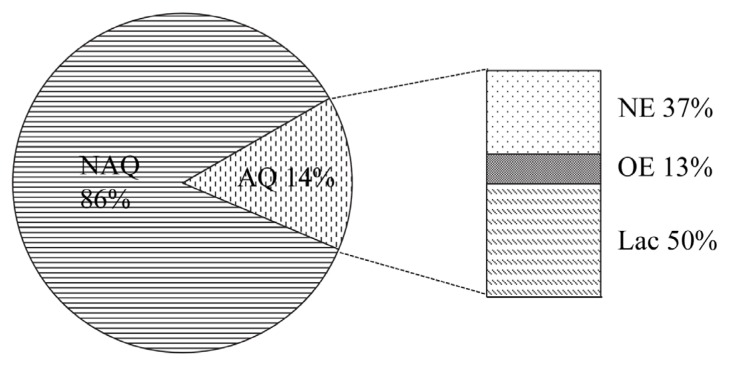
The relative abundance of phyllosphere bacteria capable of interfering AHL with different characteristics. Abbreviations: NAQ, non-AHL quencher; AQ, AHL-quencher; NE, AHL-inhibitor based on non-enzymatical interference type; OE, QQ strains degrade AHL by other quenching enzyme; Lac, QQ strains degrade AHL by AHL-lactonase.

To analyze the QS-quenching potential of the isolated bacteria originating from the tobacco phyllosphere, a degradation assay was performed with *Chromobacterium violaceum* CV026 sensor strains. Representative bioassay results are shown in Figure 1. In the bioassay, a total of 168 strains out of the 1177 isolates are capable of interfering with AHL-based QS. The initial screening of the isolates revealed that the AHL-quenchers represented 14% of the culturable leaf-associated bacteria (Figure 2). The percentage is higher than that obtained from other environments (*i.e.*, soil, rhizhosphere) in previous studies [16,17]. Only 2%–3% of the cultivable bacteria isolated from plant rhizosphere had the AHL-degrading activities [17]. Moreover, the screening of 500 strains, originating from soil, allowed the identification of 24 active strains, representing about 5% of the isolates, against AHLs [16]. Using a novel screening strategy from soil metagenomic clone libraries also produced a lower percentage of positive clones with AHL-degrading activity [18]. Indeed, the proportion of phyllosphere bacteria capable of interfering with AHLs could be even higher for the large amount of unculturable bacteria existing. Importantly, the high percentage of 14% culturable bacteria isolated from tobacco leaves had QQ activities, indicating that leaf-associated bacteria with the ability to interfere AHLs might be prevalent in the phyllosphere. It is not surprising as this might be a survival strategy in the stressful phyllosphere environment due to the fluctuations in physical conditions and the limited and highly heterogeneous availability of nutrients [14,19]. These active epiphytes with QQ activity could obtain more nutrients via signal interference, or degrading AHLs as an energy source [20].

As previous reported, several groups of small chemicals and enzymes could potentially be used for AHLs inactivity [9]. To determine the interference strategy of the AHL-based QS by the isolates, a bioassay was prepared from overnight cultures of CV026, growing in Luria-Bertani (LB) broth in the presence of spent supernatants of the tested strains [17]. Generally, after overnight growth in LB broth on microtitre plates at 28 °C, the final pH of cultures was, in all cases, not higher than 7 and therefore the deactivation of the AHLs due to alkaline pH conditions can be eliminated [21]. These CV026 reporter strains treated by cell-free culture supernatant or heat inactivated culture supernatant of phyllosphere isolates were unable to induce violacein production, indicating that 62 strains could quench QS via a nonenzyme type, which represents a 37% of the potential isolates with QQ activity (Figure 2). Small chemicals could effectively interfere with the key process in QS based on different mechanisms. For instance, the halogenated furanones, one of the quorum-sensing signal mimics, can competitively bind to the AHL receptor [22]. While, volatile organic compounds produced by the rhizospheric strain *Serratia plymuthica* could significant suppression of transcription of AHL synthase genes [23]. The mechanisms of the isolates to inactive AHL based on nonenzyme factors in the present study will be unveiled in the future.

The CV026 sensors could induce violacein production, treated by the supernatant of the isolate, but not work when treated with the inactivated culture supernatant, showing the presence of enzymatic degradation activity of the isolates. Somewhat expectedly, the potential contribution of enzymatic degradation of AHLs by the isolates reached almost 63% among the total isolates active against AHLs (Figure 2). In addition, the recovery of degraded *N*-(3-oxohexanoyl)-l-homoserine lactone (3OC6-HSL) by acidification, and visualized by CV026 indicated the presence of putative AHL-lactonase activity. More than 79% of the QQ strains with the ability to enzymatically degrade AHLs have lactonase activity (Figure 2). Despite different QQ strategies, enzymatic degradation of QS signal molecules (AHLs) has been investigated intensively [9]. Several types of enzymes, including AHL-lactonase, AHL-acylase, AHL-deaminase, and paraoxonases, have been shown to possess an ability to degrade AHL signals [24,25]. AHL-lactonase, which hydrolyses the homoserine lactone ring, is independent of the length and substitutions in the AHL signal molecule [11,26]. This might be one reason of the high frequency of the isolated QQ bacteria with AHL-lactonase activity.

### 2.2. Identification and Characterization of Bacteria with AHL-Degrading Activity

**Figure 3:**
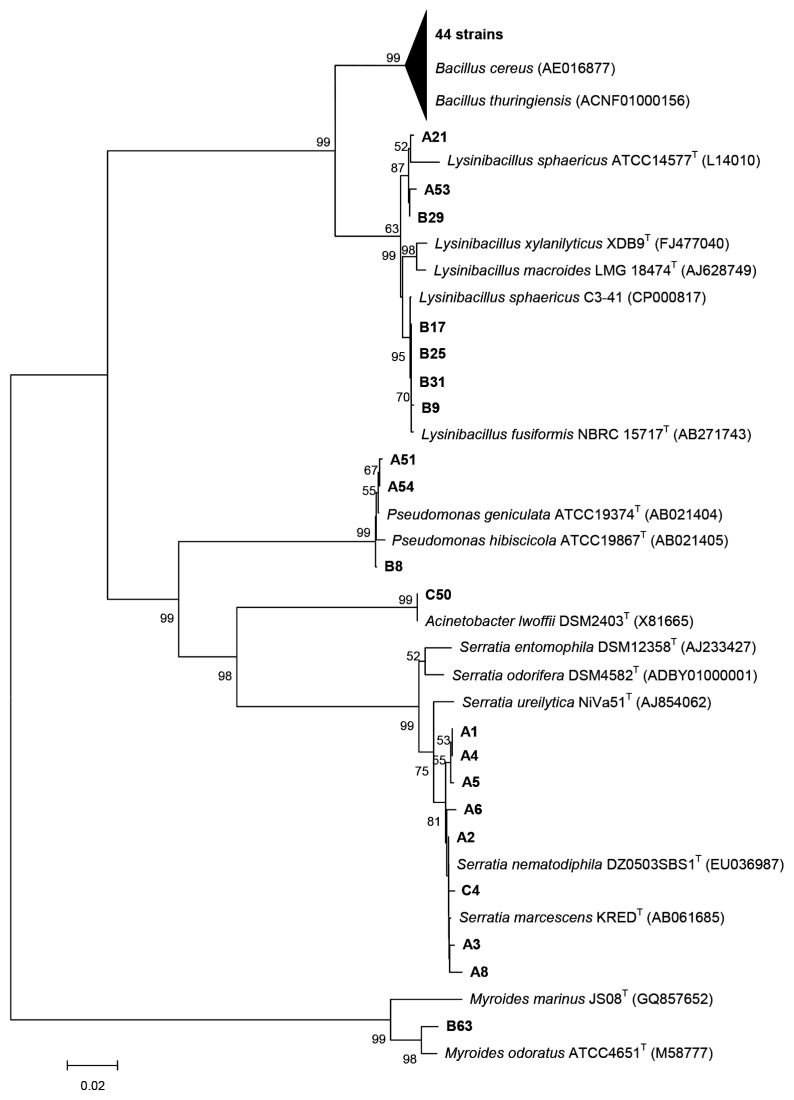
Phylogenetic relationship of the AHL-degrading phyllosphere isolates. The tree was constructed using the neighbor-joining method based on the 16S rRNA gene sequences of the isolates and reference taxa. The bacterial isolates in this study were shown in bold type. Accession numbers are indicated after the name of the reference sequences. The values represent the relative proportions that a branch appeared in 1000 bootstrap replications. Scale bar, 0.02 relative sequence divergence.

A deeper analysis of the isolated active strains with AHL-lactonase activity, including the diversity and the phylogenetic position, was performed based on the sequences of the 16S rRNA gene. Sequence comparison allowed the taxonomic affiliation and the positioning of the phyllosphere AHL-degrading strains in a phylogenetic tree (Figure 3). The Firmicutes represented approximately 75% of these AHL-degrading bacteria, while the remaining isolates were mainly Gammaproteobacteria. Bacteria capable of inactivating AHLs enzymatically are distributed among diverse taxonomic groups including Alpha-, Belta-, Gammaproteobacteria, Firmicutes, and other phyla [10]. Notably, the AHL-degrading strains were mainly restricted to the Firmicutes and Gammaproteobacteria, which accounted for a small portion of the phyllosphere bacteria communities [27]. In the Firmicutes, the AHL degraders isolated from leaf consisted in bacteria, mainly belonging to *Bacillus*. These results are in agreement with the data that lactonase activity seems to be widespread in the genus *Bacillus* [26,28]. Lactonase (AiiA) homologues have been identified in *B. cereus* and *B. thuringiensis*, which have the ability to degrade AHL [28]. *B. thuringiensis* is well known as the most widely used microbial insecticide and is environmentally safe. Recently, *B. thuringiensis* was proven as a potential biocontrol reagent to control plant pathogens for its effective suppression of the virulence of *E. carotovora* through signal interference [29]. We isolated seven phyllosphere strains affiliating with the *Lysinibacillus* genus as another major Firmicutes group capable of degrading AHL. *Lysinibacillus* has been isolated from plant tissues and are used as potential biological control agents for diseases that affect cacao [30]. To the best of our knowledge, AHL-degrading bacteria belonging to *Lysinibacillus* genus have not been reported.

The majority of the gammaproteobacterial strains with AHL-degrading activity (eight isolates) are closely affiliated with the genus *Serratia*, whereas the remaining groups, *Pseudomonas* and *Acinetobacter*, comprised three and one strain, respectively. The *Serratia* genus is found in a broad range of habitats, such as soil, water, and plants [31,32]. Akutsu and colleagues reported that a *Serratia marcescens* strain isolated from the tomato phylloplane was very effective as a biological control agent [33]. Physiological processes regulated by AHLs in *Serratia* species have been well documented, including biocontrol against plant pathogens and promotion of plant growth [34,35]. However, the leaf dwelling *Serratia* strains in the present study did not produce AHLs (data not shown), but degrade AHLs. Three gammaproteobacterial isolates, A51, A54, and B8, closely related to *Pseudomonas geniculata*, appear to be new AHL-degraders. Members of *Pseudomonas geniculata* have already been identified as aromatic compound degraders [36]. *Acinetobacter lwoffii*, which showed high similarity to strain C50, has not been reported as an AHL-degrader. As for the genus *Acinetobacter*, the strains isolated from cucumber and ginger rhizosphere have been reported to degrade AHLs, respectively [37].

Previously isolated AHL degraders from soil exclusively consisted in Gram-positive bacteria [28], whereas those from phyllosphere, in our study, encompassed both Gram-positive and Gram-negative AHL-degrading bacteria. As proposed in previous studies, this might be as a function of the environmental origin of the samples [17]. In the case of the phyllospere isolates capable of inactivating AHLs with lactonase activity, almost 25% of the isolates belonged to Gram-negative bacteria. The Gram-negative bacteria isolated from tobacco phyllosphere exhibiting AHL-degrading activity distribute in diverse genus including the genus *Serratia*, *Pseudomonas*, *Acinetobacter*, and *Myoides*. Some Gram-negative bacteria, such as the *Pseudomonas* species have been used as biocontrol reagents [38]. However, these candidate biocontrol strains did not show effective plant protection in large-scale applications because these Gram-negative bacteria are readily affected by environmental stresses [15]. Many Gram-positive bacteria, such as the *Bacillus* species, attracted more attention in the field of biocontrol due to their specialized competences [39]. The large portion (75%) of the isolated Gram-positive leaf-associated bacteria in the present study may broaden their agricultural applications as potential biocontrol agents for suppressing tobacco pathogens. In addition, these isolated bacteria have the ability to colonize the tobacco plant and are suitable to adapt to the phyllosphere microenvironment.

**Figure 4:**
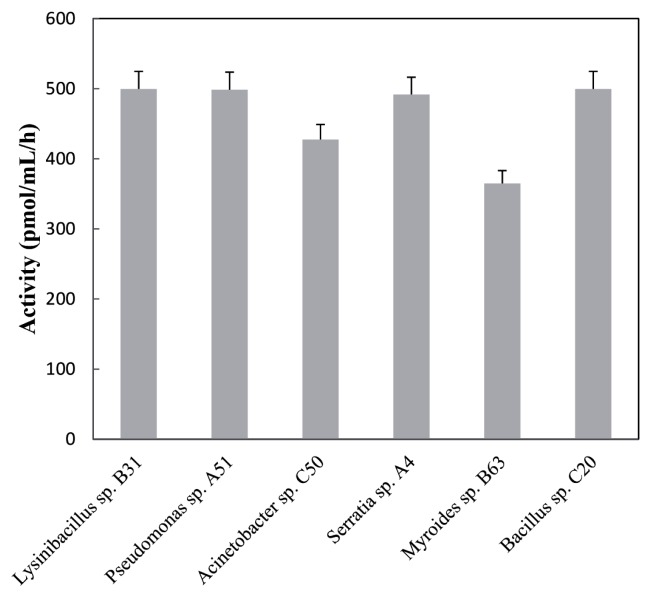
AHL inactivation analysis of the representative phyllosphere isolates. The activities are expressed in picomoles of 3OC6-HSL inactivated per hour per volume of bacterial culture. Bars indicate standard deviation values of the replicates.

The AHL-degrading activity of the representative strain with the highest relative activity among divers genus is shown in Figure 4. A comparative analysis of enzymatic activity revealed that isolates of *Bacillus*, *Lysinibacillus*, and *Pseudomonas* possessed a higher AHL-degrading activity at around 500 pmol/h/mL, while that of the *Myroides* sp. B63 was only 360 pmol/h/mL. Similarly, leaf-associated strain LPC029, isolated from *Gmelina arborea* Roxb., has an AHL-degrading activity with 400 pmol/h/mL against 3OC6-HSL [40]. In the lactonase family, AiiA lactonase is the first and most well-studied group [11,41]. Given the AiiA-like lactonases are widespread in bacteria [10], AHL-lactonase genes of the isolated phyllosphere bacteria in the present study were amplified using the *aiiA*-specific primers. However, *aiiA* genes failed to amplify in all the representative strains, except for *Bacillus* sp. C20 (Table 1). Although AiiA lactonase homologs have been characterized in a wide range of Gram-positive and Gram-negative bacteria [10], the divergent coding sequence made it is difficult to cover by specific primers. Interestingly, members of the genus *Pseudomonas* usually degrade AHLs by AHL-acylase [20,42], whereas leaf-associated *Pseudomonas* sp. A51 has a lactonase activity. The underlying mechanism of these quenching phenomena remains to be investigated in future studies.

## 3. Experimental Section

### 3.1. Isolation of Leaf Associated Culturable Bacteria

Leaf samples were randomly collected from tobacco plants (*Nicotiana tabacum* L.), at different locations, grown in a greenhouse. To isolate the leaf-associated bacteria, ten grams of mixed leaf samples were placed in sterile Erlenmeyer flasks with 30 mL 0.1 M potassium phosphate buffer (pH 7.0), using axenic procedures, and sonicated for 10 min [43]. Subsequently, the mixture was then gently vortexed for 15 min on a flask shaker. The resulting suspension was briefly centrifuged at 800 g to remove leaf debris. Serial dilutions of the final phyllosphere suspension were spread onto culture agar plates (1% tryptone, 0.5% yeast extract, and 5% sterile leaf juice) supplemented with cycloheximide (0.1 g L^−1^) to suppress fungal growth [43]. The inoculated plates were incubated at 30 °C for 48 h to culture leaf-associated bacteria. Purified isolates were stored at −80 °C in LB liquid broth with 30% glycerol until further processing.

### 3.2. Screening for Bacteria with QQ Activity

Phyllosphere bacterial isolates with the ability to degrade AHLs were screened by the bioassay method. The sensor strain *C. violaceum* CV026, producing the purple pigment violacein in the presence of short- to medium-chain AHLs [44], was used for the bioassay. Preliminary screening methods involved cultivation of sensor strains and phyllosphere bacteria at 30 °C until they grew to the exponential phase. The leaf strains isolated were grown overnight in 4 mL of LB broth including 10 μM 3OC6-HSL at 30 °C in a rotary shaker. One hundred microliters of cell-free supernatant was mixed with an equal volume of *C. violaceum* CV026 fresh cultures. Any residual AHLs were demonstrated by the activation of the biosensors [44]. To determine whether the AHL inactivation was due to enzymatic reaction, the heat inactivation method described by Dong *et al.* was used [28]. Briefly, the fresh culture of the positive isolate was treated in boiling water bath for 10 min. The treated and the fresh culture were incubated with 3OC6-HSL for different times. The presence of a QQ enzyme was confirmed by loss of AHL-degrading activity after boiling [28]. Further analysis for their capacity to degrade AHL by putative lactonase was performed using a slightly modified procedure [25]. Briefly, 10 μL of 1 M HCl was added into 90 μL of the supernatant of the degradation reaction mixture. After acidification overnight, the mixture was neutralized by addition of phosphate buffer (pH 7). The restored AHL was assessed using the above-mentioned bioassay. Quantitative analysis of AHL-inactivating enzyme activity was performed using a bioassay slice procedure. At the sampling timepoint, the reaction mixture was heat-inactivated. Subsequently, 5 μL of the heat-inactivated supernatant was loaded on the end of an LB agar slice, on which 0.5 μL of a fresh CV026 culture was progressively spotted along the bioassay slice. After incubation, the diffusion distance of the color colony from the origin, in each agar slice, was measured.

### 3.3. PCR Amplification and Sequence Analyses

DNA was extracted from pure cultures using a bacterial genomic DNA isolation kit (Takara, Dalian, China) according to the manufacturer’s instructions. The bacterial 16S rRNA gene was amplified based on a template of the purified genomic DNA with universal primers 27F (AGAGTTTGATCMTGGCTCAG) and 1525R (AAGGAGGTGWTCCARCC) [43]. PCR amplification was performed in an Eppendorf mastercycler gradient PCR machine, using ExTaq DNA polymerase (Takara, Dalian, China). Amplification conditions were identical for all samples, with an initial denaturation at 94 °C for 5 min, followed by 30 cycles of 94 °C for 30 s, 58 °C for 30 s, and 72 °C for 2 min; and a final elongation at 72 °C for 10 min. The *aiiA* gene was amplified with ExTaq DNA polymerase and the aiiAF16 and aiiAR705 primers, according to the procedure in previous studies [17]. The PCR products with correct size checked on 1% agarose gel by electrophoresis were purified with the EZNA cycle pure kit (Omega Bio-Tek Inc., Norcross, GA, USA) for subsequent use.

Purified amplicons were cloned using the pGEM-T vector (Promega, Madison, WI, USA) with *Escherichia coli* DH5α chemically competent cells (Takara) in accordance with the manufacturer’s instructions. Colony PCR was performed with the primers T7F and SP6R according to protocol [45]. Sequencing of positive clones was performed with the standard primer T7F on an ABI PRISM 3730 sequencer (Applied Biosystems, Foster, CA, USA). Raw sequence data were assembled and trimmed using DNAStar software Version 7.1 (Madison, WI, USA). Chimeric sequences were removed following evaluation by the Mallard program [46]. The 16S rRNA gene sequences were compared with the sequences in the GenBank database and EZtaxon database to obtain the nearest phylogenetic neighbors [47]. Phylogenetic trees were constructed using neighbor-joining algorithm, implemented in MEGA version 5.0 (Tempe, AZ, USA), with 1000 bootstrap replicates [48]. The sequences for 16S rRNA genes generated in this study have been submitted to the GenBank database and assigned accession numbers KF114398 to KF114462.

## 4. Conclusions

A wide range of QQ bacteria associated with the tobacco leaf have been isolated and characterized as belonging to the genus *Bacillus*, *Lysinibacillus*, *Serratia*, *Pseudomonas*, *Acinetobacter*, and *Myoides*. The high percentage and diversity of QQ phyllosphere bacteria isolated in the present study indicated that QQ seems to be a usual strategy for epiphytes to survive in a phyllosphere microenvironment. These indigenous leaf-dwelling QQ strains may broaden their agricultural applications as potential biocontrol agents to suppress tobacco pathogens. Moreover, these QQ bacteria mainly have the ability to degrade AHL by lactonase enzyme. Further studies will focus on the molecular basis of cultivable leaf-associated bacteria with AHL-lactonase activity.

## Figures and Tables

**Table 1:** Identification and characterization of the representative phyllosphere isolates with lactonase activity.

Strain	Related bacteria	Similarity (%)	C6-HSL	3OC6-HSL	*aiiA*
B31	*Lysinibacillus fusiformis* NBRC15717	99.9	+++	+++	NA
A51	*Pseudomonas geniculata* ATCC19374	99.9	+++	+++	NA
C50	*Acinetobacter lwoffii* DSM2403	99.5	++	++	NA
A4	*Serratia marcescens*	99.8	+++	+++	NA
B63	*Myroides odoratus* ATCC4651	98.4	+	+	NA
C20	*Bacillus cereus* ATCC14579	99.9	+++	+++	+

Capacity to inactive C6- and 3OC6-HSL is shown, as well as the analysis of the *aiiA* gene of the isolate. +, low enzyme activity (incompletely degraded 10 μM AHL within 12 h); ++, intermediate enzyme activity (completely degraded 10 μM AHL within 12 h); +++, high enzyme activity (completely degraded 10 μM AHL within 6 h); NA, not amplified.
